# Disparities in Timely Access to Certified Stroke Care Among US Census Tracts, by Prevalence of Health Risk Factors

**DOI:** 10.5888/pcd22.240429

**Published:** 2025-07-03

**Authors:** Linda Schieb, Joshua Tootoo, Melissa Fiffer, Michele Casper, Dominique Pierre Zephyr, E. Bradshaw Bunney, Marie Lynn Miranda

**Affiliations:** 1 Division for Heart Disease and Stroke Prevention, Centers for Disease Control and Prevention, Atlanta, Georgia; 2 Children’s Environmental Health Initiative, University of Illinois Chicago; 3 College of Medicine, University of Illinois Chicago; 4 Departments of Pediatrics and Mathematics, Statistics, and Computer Science, University of Illinois Chicago

## Abstract

**Introduction:**

Timely access to stroke care reduces death and disability due to stroke. Studies have investigated disparities in access by sociodemographic characteristics but not comorbidity prevalence. We used updated data to assess both types of disparities in drive times to certified stroke centers nationwide.

**Methods:**

We conducted a cross-sectional spatial analysis of drive time from each contiguous US census tract (N = 72,517), using population-weighted centroids, to any certified stroke care (n = 1,825) or advanced (ie, endovascular-capable) stroke care (n = 426), using 2022 data from multiple state and nationwide databases. We compared median comorbidity prevalence and sociodemographic characteristics for census tracts within versus beyond a 60-minute drive time, using US Centers for Disease Control and Prevention PLACES 2020 data.

**Results:**

Median (interquartile range) drive time was 11.8 (7.6–21.6) minutes to any certified stroke care, and 23.0 (12.6–53.9) minutes to advanced stroke care. Approximately 20% of the US adult population (n = 49 million) resided in census tracts beyond a 60-minute drive from advanced stroke care; most (65%) were rural. Census tracts more than 60 minutes from advanced stroke care had significantly higher prevalence of stroke, high blood pressure, coronary heart disease, high cholesterol, diabetes, chronic kidney disease, fair or poor self-rated health status, smoking, and obesity. They also had higher poverty rates, lower educational attainment, lower median income, and higher proportions of non-Hispanic White people and people older than 65 years.

**Conclusion:**

Residents in census tracts lacking timely access to stroke care have higher prevalence of health risk factors. The results highlight areas where education, telehealth infrastructure, and facility placement could improve stroke systems of care.

**Summary**

**What is known on this topic?**

Research has identified disparities in geographic access to stroke care in the US by sociodemographic characteristics. We used nationwide stroke center data from 2022 to examine disparities in access by prevalence of multiple stroke risk factors.

**What is added by this report?**

Using population-weighted centroids, we found that approximately 20% of the US adult population (N = 49 million) resided in census tracts beyond a 60-minute drive from advanced stroke care. Furthermore, census tracts lacking timely access have higher prevalence of health risk factors.

**What are the implications for public health practice?**

The results highlight areas where education, telehealth infrastructure, and facility placement could improve stroke systems of care.

## Introduction

Stroke is the fifth leading cause of death in the United States (1), responsible for 41.1 deaths per 100,000 population (2). People with cardiometabolic comorbidities, including previous stroke, are at greater risk (1,3). Minimizing the time from stroke onset to treatment is critical to improving stroke outcomes (4). Two key time determinants are 1) symptom recognition and 2) stroke center proximity (5). Prior research has begun to uncover geographic patterns in access to stroke care, yet this is the first study to examine disparities in drive time by prevalence of multiple stroke risk factors. Some studies have characterized demographic disparities in timely access to stroke care (6–8); few have investigated disparities in stroke risk factors, beyond prior stroke or location within a Stroke Belt state (9,10).

We used an updated data set compiled from multiple state and nationwide certification sources, whereas earlier studies considered access to subsets of stroke facilities (eg, only looking at national certification databases or 1 to 2 levels of stroke care) (11,12) or subregions of the US (8,13–15). One recent study examined nationwide geographic access using national and state certification databases at the census tract level (16). However, the authors presented only driving distances, whereas drive times are critical, as they vary substantially with topography and road density.

We used geospatial analysis to estimate census tract–level drive time to stroke centers in the contiguous US, using national and state certification databases to identify all levels of certified stroke care. We assessed both comorbidity prevalence and sociodemographic characteristics of populations residing within versus beyond a 60-minute drive to these facilities. We hypothesized that rural tracts and those with higher prevalence of health risk factors would lack timely stroke care access.

## Methods

### Study design

This cross-sectional analysis estimated drive time from each census tract in the contiguous US to the nearest certified stroke care facility. Alaska and Hawaii were excluded due to their higher frequency of non–road-based travel (17). Comorbidity prevalence and sociodemographic characteristics were compared between census tracts within and beyond a 60-minute drive time from certified stroke centers. A 60-minute drive time threshold helps meet the imperative to treat stroke patients within 3 to 4.5 hours of initial onset of symptoms, including the clinical goal of a 30-minute hospital door-to-needle administration of intravenous thrombolytic therapy (IVTT) (18). This measure was the most common threshold used in recent studies of timely access to stroke care (5,10,19,20). In mapped results, we also included 30-minute drive time cut-offs.

Institutional review board approval and informed consent were deemed unnecessary because the health data used in this research was all nonidentifiable public data. The study adhered to the Strengthening the Reporting of Observational Studies in Epidemiology (STROBE) guidelines for cross-sectional studies.

### Data sources

A nationwide data set of certified stroke centers (N = 1,825), including address-level locations and certification type, was built from nationwide and state certification databases. Data were provided to the Centers for Disease Control and Prevention (CDC) or accessed from official websites of the Joint Commission (on February 23, 2022), Det Norske Veritas (DNV Holding AS) (on February 24, 2022), and the Accreditation Commission for Health Care (on January 17, 2022). Of the 6 states indicating an established state-specific designation for stroke centers, 4 (Maryland, Massachusetts, Missouri, New Jersey) had data and criteria publicly available for download (accessed March 8, 2022) (21). For the 16 disagreements between certification types for the same facility across certifying bodies, we retained the highest level of certification. We eliminated 56 duplicate records.

The certification types were Acute Stroke Ready Hospital (ASRH), Primary Stroke Center (PSC), Thrombectomy-Capable Stroke Center (TSC), and Comprehensive Stroke Center (CSC). Briefly, ASRHs are smaller community hospitals that use established protocols for acute stroke evaluation and treatment (including telemedicine). Patients must be transferred to higher-level facilities for IVTT (19). PSCs provide care in a dedicated stroke unit, including diagnosis of ischemic stroke and administration of IVTT. TSCs additionally offer mechanical intervention via endovascular thrombectomy. CSCs use expertise in stroke neurology, critical care, and neurosurgery to manage the most complex ischemic and hemorrhagic stroke cases. We grouped certification types into 2 categories to represent the relative level of care, from least to most exclusive: 1) any certified stroke care (ASRH, PSC, TSC, CSC) and 2) advanced stroke care (TSC, CSC).

Prevalence of self-reported stroke and stroke risk factors was obtained at the census tract level for US adults aged 18 years or older (N = 72,517 census tracts) from CDC’s PLACES data (2020) (22). Rurality of census tracts was defined based on the Health Resources & Services Administration’s (HRSA) Rural Health Grants Eligibility Analyzer data (2022) (23). Census tract–level sociodemographic variables, including race and ethnicity, age group, and measures of socioeconomic status, were obtained from the American Community Survey 5-year estimates, 2013–2017 (24).

### Spatial/network analysis

We estimated drive times from the population-weighted centroid of a census tract to the nearest stroke care facility in several steps. First, population-weighted centroids (PWCs) were obtained for census tracts based on the 2010 Decennial Census boundaries (to align with PLACES data, which relies on 2010 boundaries) (25). Second, longitude and latitude were used to map these census tract PWCs in coordinate space and project them to the Global Coordinate System North American Datum of 1983 (GCS NAD83). Third, to minimize positional error or distortion, tract PWCs were reprojected to match the World Geodetic System (WGS 84) projection, which is used in Esri’s StreetMap Premium 2022 Release 2 network data set (SMP22r2) (Esri). Fourth, we improved our ability to estimate drive times by using the closest network junction for each tract PWC as the starting point, resulting in a more than 99% solve rate for analyses. And fifth, we calculated drive times for passenger vehicles, assuming no traffic congestion and that vehicles follow the speed limit.

### Outcome

Primary outcomes were 1) drive time to any certified stroke care or advanced stroke care; 2) the proportion of individuals who live in census tracts more than 60 minutes from certified stroke care; 3) the comorbidity burden of census tracts beyond 60 minutes to certified stroke care; and 4) the sociodemographic characteristics of those census tracts. In addition, we assessed and mapped drive time to CSCs on their own.

### Statistical analysis

Median and interquartile range (IQR) drive times to stroke care were calculated for census tracts. For each level of stroke care, census tracts were considered more than 60 minutes away if the minimum calculated drive time from the census tract PWC was greater than 60 minutes to the closest stroke center. Due to skewness in sociodemographic and comorbidities variables, we used Mann–Whitney U tests to compare characteristics of census tracts within versus beyond a 60-minute drive time; *P* < .05 for a 2-sided test was considered significant. All analyses were coded and conducted in SAS version 9.4 (SAS Institute) and recoded and repeated in R version 3.6.2 (R Foundation) to ensure accuracy and reproducibility.

## Results

This study calculated stroke drive times from census tracts in the contiguous US (N = 72,517) to 1,825 facilities with any certified stroke care (ASRH, PSC, TSC, or CSC), and 426 facilities offering advanced stroke care (TSC or CSC). Overall, the median (IQR) drive time to any certified stroke care was 11.8 (7.6–21.6) minutes (Table 1). In contrast, the median (IQR) drive time to advanced stroke care facilities was nearly double at 23.0 (12.6–53.9) minutes.

**Table 1:** Summary of Geographic Proximity to Certified Stroke Care for Adults (N = 245,641,071) in 72,517 US Census Tracts, by Level of Care

Characteristic	Any certified stroke care (ASRH, PSC, TSC, or CSC)	Advanced stroke care (TSC or CSC)
**Overall**		
No. of facilities	1,825	426
Drive time, median (IQR), min	11.8 (7.6–21.6)	23.0 (12.6–53.9)
Distance, median (IQR), mi	5.9 (3.0–15.0)	15.5 (6.0–46.8)
**Drive time >60 min**		
Census tracts, no. (%)	4,352 (6.0)	16,081 (22.2)
Rural, %	90.7	65.0
Population aged ≥18 y, no. (%)	11,601,897 (4.7)	49,381,586 (20.1)

Abbreviations: ASRH, acute stroke ready hospital; CSC, comprehensive stroke center; IQR, interquartile range; PSC, primary stroke center; TSC, thrombectomy-capable stroke center.

Approximately 5% of the contiguous US adult (aged ≥18 y) population lived in census tracts more than 60 minutes from any certified stroke care facility. (Table 1). These census tracts were predominantly rural (approximately 91%). An estimated 20% of the adult contiguous US population (approximately 49 million people) resided beyond a 60-minute drive from facilities for advanced stroke care (TSC or CSC). Rural geographic regions constituted approximately 65% of the census tracts without timely (60-minute) access to advanced stroke care.

Populations living in census tracts more than 60 minutes away from a stroke care facility showed significantly higher comorbidity prevalence (Table 2). The median prevalence of stroke was 3.5% (3.0%–4.2%) in these census tracts, compared with 2.8% (2.2%–3.5%) for census tracts within a 60-minute drive (*P* < .001). The significantly elevated prevalence in underserved census tracts persisted across all other comorbidities and stroke risk factors studied, including high blood pressure (35.7% vs 30.8%), diabetes (11.6% vs 9.8%), smoking (19.7% vs 15.8%), and obesity (36.2% vs 31.9%) (*P* < .001).

**Table 2:** Disparities in Comorbidity Prevalence Among US Census Tracts, by Drive Time to Certified Stroke Care^a^

Comorbidity, %	Any certified stroke care (ASRH, PSC, TSC, or CSC)			Advanced stroke care (TSC or CSC)		
	>60 Min, median (IQR)	≤60 Min, median (IQR)	*P* value	>60 Min, median (IQR)	≤60 Min, median (IQR)	*P* value
Stroke	3.7 (3.2–4.3)	2.9 (2.3–3.7)	<.001	3.5 (3.0–4.2)	2.8 (2.2–3.5)	<.001
High blood pressure	35.9 (32.5–40.1)	31.6 (27.4–36.5)	<.001	35.7 (31.9–40.2)	30.8 (26.8–35.4)	<.001
Coronary heart disease	7.8 (6.8–8.9)	5.9 (4.8–7.3)	<.001	7.4 (6.3–8.5)	5.7 (4.6–6.9)	<.001
High cholesterol	35.2 (32.3–37.6)	31.9 (28.9–34.8)	<.001	34.6 (31.8–36.9)	31.4 (28.6–34.2)	<.001
Diagnosed diabetes	11.8 (10.2–13.7)	10.1 (8.3–12.5)	<.001	11.6 (9.8–13.6)	9.8 (8.1–12.1)	<.001
Chronic kidney disease	3.4 (3.0–3.8)	2.8 (2.4–3.4)	<.001	3.3 (2.8–3.7)	2.7 (2.3–3.3)	<.001
Fair or poor self-rated health status	16.5 (13.5–20.9)	14.3 (10.8–19.2)	<.001	16.6 (13.3–21.2)	13.7 (10.4–18.6)	<.001
Smoking	19.2 (16.6–22.5)	16.6 (12.6–21.0)	<.001	19.7 (16.5–23.4)	15.8 (12.1–20.3)	<.001
Obesity	35.4 (31.7–38.8)	32.9 (27.9–37.5)	<.001	36.2 (32.5–39.5)	31.9 (27.1–36.7)	<.001

Abbreviations: ASRH, acute stroke ready hospital; CSC, comprehensive stroke center; IQR, interquartile range; PSC, primary stroke center; TSC, thrombectomy-capable stroke center.

a *P* values from a 2-sided Mann–Whitney U test.

Census tracts lacking timely access to stroke care also demonstrated significantly larger socioeconomic vulnerabilities (Table 3). For example, census tracts beyond a 60-minute drive time to advanced stroke care facilities had a higher median percentage of the population living below the federal poverty level (15.0% [9.7%–22.6%] vs 11.5% [6.0%–20.8%], *P* < .001), as well as percentage without a high school diploma (12.1% [7.4%–18.9%] vs 9.8% [5.0%–17.8%], *P* < .001). Similarly, these census tracts had significantly lower median percentages of the population with a college education and a lower median household income.

**Table 3:** Disparities in Sociodemographic Characteristics Among Census Tracts, by Drive Time to Certified Stroke Care

Sociodemographic characteristic	Any certified stroke care (ASRH, PSC, TSC, or CSC)			Advanced stroke care (TSC or CSC)		
	>60 Min, median (IQR)	≤60 Min, median (IQR)	*P* value^a^	>60 Min, median (IQR)	≤60 Min, median (IQR)	*P* value^a^
Below poverty, %	14.9 (10.0–21.4)	12.2 (6.5–21.3)	<.001	15.0 (9.7–22.6)	11.5 (6.0–20.8)	<.001
Less than high school diploma, %	11.2 (7.4–17.5)	10.3 (5.4–18.1)	<.001	12.1 (7.4–18.9)	9.8 (5.0–17.8)	<.001
College education, %	52.2 (43.0–60.6)	58.2 (45.2–72.5)	<.001	50.9 (41.2–61.5)	60.2 (46.8–74.3)	<.001
Median household income, $	45,085 (36,483–54,125)	55,250 (40,571–76,191)	<.001	46,280 (36,719–56,790)	58,281 (41,634–80,565)	<.001
Unemployed, %	5.6 (3.3–9.0)	5.9 (3.9–8.9)	<.001	5.9 (3.7–9.0)	5.9 (3.9–8.8)	.14
NH Black population, %	0.5 (0.0–2.5)	4.3 (1.0–15.5)	<.001	1.4 (0.2–8.6)	4.8 (1.2–16.4)	<.001
Hispanic population, %	3.2 (1.1–10.3)	7.4 (2.6–20.8)	<.001	3.4 (1.2–10.1)	8.6 (3.2–23.1)	<.001
NH White population, %	86.3 (65.6–94.3)	69.6 (37.5–87.1)	<.001	84.3 (62.4–94.1)	66.4 (33.0–84.9)	<.001
Age ≥65 years, %	19.1 (14.8–23.4)	14.5 (10.3–18.7)	<.001	17.4 (13.4–21.2)	14.0 (9.9–18.2)	<.001

Abbreviations: ASRH, acute stroke ready hospital; CSC, comprehensive stroke center; IQR, interquartile range; NH, non-Hispanic; PSC, primary stroke center; TSC, thrombectomy-capable stroke center.

a *P* values from a 2-sided Mann–Whitney U test.

Areas lacking timely access to stroke care had significantly larger older or non-Hispanic White populations, and smaller non-Hispanic Black and Hispanic populations (Table 3). For census tracts beyond a 60-minute drive time to advanced stroke care facilities, the median percentage (IQR) of adults aged 65 years or older was 17.4% (13.4%–21.2%), compared with 14.0% (9.9%–18.2%) for census tracts within a 60-minute drive time (*P* < .001).

**Figure 1:**
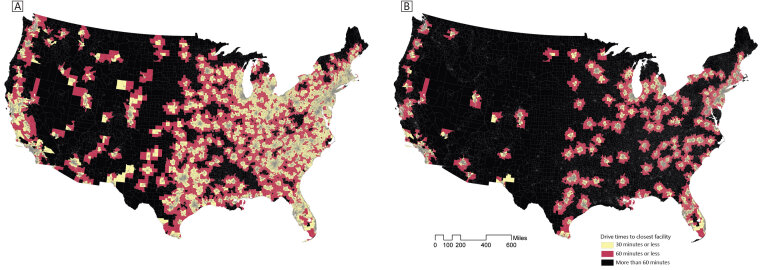
Maps of census tracts within versus beyond 30- or 60-minute drive time of A) any stroke care (ie, ASRH, PSC, TSC, or CSC) and B) advanced stroke care (ie, TSC or CSC). Abbreviations: ASRH, Acute Stroke–Ready Hospital; CSC, comprehensive stroke center; PSC, primary stroke center; TSC, thrombectomy-capable stroke center.

**Figure 2:**
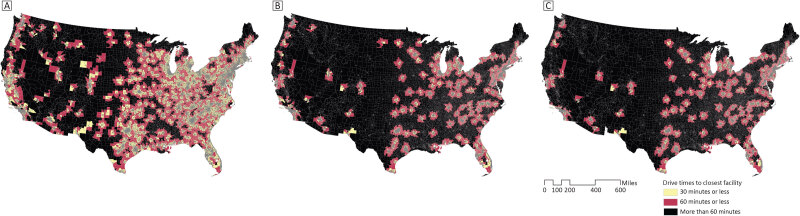
Maps of census tracts within versus beyond 30- or 60-minute drive time of A) any certified stroke care (ie, ASRH, PSC, TSC, or CSC); B) advanced stroke care (ie, TSC or CSC); and C) comprehensive stroke care (CSC) only. Abbreviations: ASRH, Acute Stroke–Ready Hospital; CSC, comprehensive stroke center; PSC, primary stroke center; TSC, thrombectomy-capable stroke center.

Maps of 30- and 60-minute drive times to certified stroke care facilities illustrated substantial geographic inequities in proximity to stroke care (Figure 1). Most census tracts along the East Coast, in the Midwest, and the Gulf Coast are located within a 30-minute or 60-minute drive to a certified stroke care facility. Louisiana, Mississippi, and Alabama stand out geographically given the lack of timely access to even basic stroke care in these communities. A strip of census tracts along the West Coast has timely access to any stroke care facility, but large swaths of the Southwest, Mountain Region, Northern Plains, and Central US lack timely access to any certified stroke care facility. The maps for drive times to advanced stroke care facilities showed even larger geographic disparities in timely access to endovascular-capable facilities. These areas were characterized by a patchwork of small clusters of census tracts within 30- or 60-minute drive times, set against the backdrop of many census tracts “in the dark” (without timely access). The supplemental map of drive times to CSCs only followed a similar pattern to the advanced stroke care (TSC or CSC) map (Figure 2).

## Discussion

One-fifth of the country’s population — more than 49 million adults aged 18 years or older — lacked timely access to advanced stroke care (TSC or CSC). Census tracts beyond a 60-minute drive time to a stroke center were more likely to have higher prevalence of stroke risk factors, including high blood pressure, coronary heart disease, high cholesterol, diabetes, chronic kidney disease, fair or poor self-rated health, smoking, and obesity. Also, census tracts beyond a 60-minute drive time to a certified stroke center were more likely than those within a 60-minute drive time to have a higher median percentage of population living below poverty, lower educational attainment level, and lower median household incomes; they also were more likely to have larger percentages of older adults and non-Hispanic White people. Geographically, large swaths of census tracts across the South, the intermountain West, and rural areas across the country lacked timely access to advanced stroke care.

Our study adds to the body of literature examining access to any certified stroke care. In 2010, there were 811 Joint Commission PSCs. Using this 2010 PSC data, Mullen et al, Khan et al, and Adeoye et al each independently found that 33%–34% of population did not have timely ground access to PSCs (5,14,26). By 2019, a study by Aldstadt et al analyzing 1,624 stroke centers certified as ASRH or PSC (nonendovascular-capable centers) estimated that the percentage of the US population beyond an hour drive from basic stroke care had dropped to 22% (20). Another study based on 2019 data estimated that less than 10% of the US population was more than 60 minutes away from an emergency department with any level of certified stroke care (27). Our updated analysis based on 2022 data from multiple nationwide and state certification databases places this estimate at just below 5%, with a total of 1,825 facilities with any level of certified stroke care.

Although the number of advanced stroke care facilities has likewise increased over time, we found that inequities in timely access persisted for a substantial proportion (one-fifth) of the US population. This finding is particularly problematic for more severe cases where patients may require mechanical intervention. In 2010, an estimated 44% of the country lived beyond a 60-minute drive from an endovascular-capable hospital (5). In 2019, 50% of the US population did not have timely ground access to the 322 stroke centers certified as TSC or CSC (endovascular-capable centers) (20). Our study demonstrates that, in 2022, there were 426 advanced stroke care facilities, and yet 20% of the population still did not have timely access to advanced stroke care (TSC or CSC), critical to improving stroke outcomes. The growth in advanced stroke centers is largely due to the conversion of existing PSCs at large academic centers, urban tertiary care centers, and large community suburban hospitals into advanced stroke centers, rather than the addition of new facilities in harder to reach rural areas.

To our knowledge, this is the first US study comparing the prevalence of stroke risk factors among communities with and without timely access to certified stroke centers, beyond examining previous stroke rates or location in a Stroke Belt state. A 2013 study of access to stroke care in South Carolina found that counties with high stroke death rates had limited or no timely access to a PSC (15). Similarly, a 2014 study estimated that 55% of the population in the Stroke Belt (Alabama, Arkansas, Georgia, Louisiana, Mississippi, North Carolina, South Carolina, and Tennessee) was beyond a 60-minute ambulance drive time of a PSC, compared with just 30% of the population in non–Stroke Belt states (10). A 2021 study in South Korea considered stroke risk factors and found that patients who lived farther from admitting hospitals were more likely to have atrial fibrillation but less likely to have other cardiometabolic issues like hypertension, diabetes, high cholesterol (hyperlipidemia), and coronary artery disease (9). In contrast, we identified significantly higher prevalence of all the cardiometabolic risk factors that we studied (high blood pressure, coronary heart disease, high cholesterol, diabetes, chronic kidney disease, fair or poor self-rated health, smoking, and obesity) in census tracts beyond a 60-minute drive time of any certified stroke care and/or advanced stroke care.

Prior studies have identified large sociodemographic disparities in geographic access to stroke centers, with the most substantial gaps in rural areas (7,10,13,14,16,26). In their analysis of proximity to PSCs in the southeastern region of the US, Khan et al reported the largest disparity to be based on rurality, with only one quarter of the rural population living within a 30-minute drive time of a PSC, compared with most (70%) of the urban population (14). A 2022 study of emergency medical services (EMS) transit time found that median ground transport was 40 minutes for rural areas versus 35 minutes for the overall population (28). Like these studies, we also noted greater disparities in timely access to stroke care in rural areas. The few previously published studies that included socioeconomic characteristics also reported more disadvantages in communities without timely access or with greater distances to stroke centers (7,8,14,16). We found that census tracts beyond a 60-minute drive time to a certified stroke center were more likely to have a higher median percentage of population living below the poverty line, lower educational attainment level, and lower median household incomes. Hospitals in low-income and rural communities have also been shown to have a lower likelihood of receiving stroke certification (29).

Consistent with existing literature and despite known disparities in stroke illness and death rates by race and ethnicity, we determined that census tracts lacking timely access to care were more likely to be predominantly non-Hispanic White (8,10). Stroke death rates are higher for non-Hispanic Black people (59.6 per 100,000) than for non-Hispanic White (39.8 per 100,000) and Hispanic (36.1 per 100,000) people (2). Accounting for hospital and population size, a 2022 study determined that the chances of stroke certification were lower for racially segregated, predominantly non-Hispanic Black communities (29), underscoring the importance of considering interactions among multiple social stressors in future studies of stroke care access. Older adults (aged ≥65 y) are particularly vulnerable (stroke death rate of 277.8 per 100,000 — more than 6 times the overall rate) and less likely to have timely access to stroke care (14,16).

This study has limitations. Our estimated drive times assumed no traffic congestion, as factoring traffic would be computationally intensive and require implementation in hourly, daily, and seasonal time steps for a national analysis. We focused on drive time for passenger vehicles, rather than EMS transport times; however, this may provide a more accurate estimate for rural areas which have relatively low use of EMS. We also did not incorporate estimates of non–road travel times, such as EMS air transport. Other studies have examined EMS transport times by ground and by air, providing a lens into understanding the contribution of EMS time to total prehospital time (including dispatch, response, scene, and transport) (5,20,28). As for statistical methods, we applied Mann–Whitney U tests to compare distributions between groups and did not use any multivariable regression analyses. We acknowledge that other factors and potential confounders such as the speed of recognition of stroke symptoms by the patient, caregiver, and/or bystanders and the performance of the stroke care facility are important contributors to prompt stroke treatment. In addition, a hospital’s stroke certification level may change over time. We identified hospital stroke care certification levels as a snapshot in 2022 and captured only the level at that time. We also may have overlooked some state certifications. A recent study has generated a national database of certified stroke centers, but it relied on older (2018) data (30). Our results may differ from other studies because Alaska and Hawaii, states with high frequency of air and water EMS transport, were not included. Finally, the comorbidity data derived from the PLACES data relies on self-reported data through the Behavioral Risk Factor Surveillance System.

Our study has many strengths, including the use of drive times rather than simple distances, and the innovation in linking data sets to provide a full picture of communities by their access to stroke care. In addition, the study was implemented at a relatively fine geographic resolution of the census tract level. We used population-weighted centroids to measure distances more accurately from where people actually reside. We also examined the prevalence of multiple stroke risk factors at the census tract level in addition to measures of sociodemographic characteristics. Using multiple national certification bodies and state databases, we constructed a nationwide database of stroke facilities across all levels of certified stroke care. This allowed us to compare access to the different types of stroke care facilities and differentiate between those who have access to any type of facility versus those who have access to the most advanced stroke care.

This study provides insights for clinical and public health professionals working to improve stroke systems of care. As emphasized in a recent consensus statement from major national organizations in the fields of stroke, neurology, and EMS (31), region-specific stroke systems of care are needed to address differences in resources, hospital certifications, geography, population density, and community knowledge of stroke symptoms. Plans for addressing a range of recommendations for stroke systems of care, outlined in the 2019 American Stroke Association report (19) and the 2021 complementary consensus statement (31), include raising awareness of stroke signs and symptoms and the need to call 911, enhanced primary and secondary stroke prevention activities, informed EMS destination plans, coordinated interhospital transfer policies, quality improvement programs, and data collection priorities. Local patterns in timely access to stroke care by stroke certification status, and the associated comorbidities and sociodemographic characteristics, can help inform those plans.

Many states have already implemented programs and policies to improve stroke systems of care (32). State laws with the best evidence for positive health impact and an associated economic impact include EMS triage and transport to the most appropriate stroke facility, air medical transport to the most appropriate stroke facility, interfacility transfer to the most appropriate stroke facility, nationally certified primary stroke centers, state standards for stroke centers, and telestroke to initiate treatment on-site (33).

CDC’s Paul Coverdell National Acute Stroke Program funds competitively selected state health departments to collect, measure, and track data to improve the quality of care for stroke patients (34). Health department staff work with EMS agencies and hospitals to improve the efficiency and quality of care and have developed a Stroke Systems of Care Framework, including resources to guide others (35).

In conclusion, substantial inequities in timely access to basic and advanced stroke care exist, with higher prevalence of stroke comorbidities and greater sociodemographic disadvantages in communities that are further than a 60-minute drive to a stroke center. Mapping timely access to stroke care by level of stroke center certification highlights the communities that would benefit most from systematic improvements in stroke systems of care. These improvements include educating for awareness of stroke signs and symptoms, expanding telestroke care and regional stroke transport programs, and identifying and treating stroke risk factors.
